# Role of triglyceride-glucose index in metabolic assessment of sarcoidosis patients

**DOI:** 10.1007/s11739-024-03609-4

**Published:** 2024-04-24

**Authors:** Luigi Rizzi, Chiara Coppola, Yaqob Hbaidi, Riccardo Russo, Lucilla Crudele, Antonio Moschetta, Patrizia Suppressa

**Affiliations:** Department of Internal Medicine, and Rare Diseases Centre “C. Frugoni”, University Hospital of Bari, Piazza Giulio Cesare 11, 70124 Bari, Italy

**Keywords:** Rare disease, Sarcoidosis, TyG index, Metabolic syndrome, Cardiovascular risk

## Abstract

Sarcoidosis is a rare granulomatous disease that can affect any organ. It leads to an increased risk of metabolic syndrome and insulin resistance, due to biochemical pathways involved in low-grade inflammation in both diseases. The aim of our retrospective case–control study was to evaluate the utility of triglyceride-glucose (TyG) index, a surrogate of insulin resistance, for metabolic assessment of sarcoidosis patients. A cohort of 90 sarcoidosis patients and a cohort of 90 control subjects were enrolled. Clinical, anamnestic, and biochemical data were collected. Results showed that TyG index values were higher in the sarcoidosis cohort than in the control group (*p* < 0.001), even after excluding the influence of diabetes and metabolic syndrome (*p* = 0.018). In the sarcoidosis cohort, TyG index was not correlated with clinical phenotyping (*p* = 0.358), gender (*p* = 0.139), radiological stage (*p* = 0.656), glucocorticoids cumulative dose (*p* = 0.682) or treatment regimen (*p* = 0.093), while significant positive correlations with waist circumference (*p* < 0.001), systolic and diastolic pressure (*p* = 0.041 and *p* = 0.029, respectively), Framingham score (*p* = 0.007) were found. Receiving operating characteristics curve analysis identified a TyG index optimal cut-off value of 8.64 (66.7% sensitivity, 77.8% specificity, area under the curve -AUC- 75%, 95% confidence interval -CI- 65–85, *p* < 0.001) to detect metabolic syndrome and a cut-off value of 8.69 (64.1% sensitivity, 70.6% specificity; AUC 67%, 95% CI 55–78, *p* = 0.007) to detect an intermediate cardiovascular risk according to Framingham risk score. Concluding, TyG index can be considered a useful tool for the metabolic assessment of sarcoidosis patients, given its capacity to predict metabolic syndrome and cardiovascular risk.

## Introduction

Sarcoidosis is a rare chronic multisystemic disease of unknown etiology characterized by the presence of non-necrotizing granulomas in the involved organs. Respiratory system is one of the most frequently affected sites, although any organ can be virtually involved [1]. The prevalence of sarcoidosis varies depending on the geographical area, ranging from 1 to 5 per 100,000 in South Korea, Taiwan and Japan to 140–160 per 100,000 in Sweden and Canada; the incidence is likewise variable, ranging from 11.5 per 100,000 in Sweden to 0.5–1.3 per 100,000 in East Asia [2]. Clinical presentation is heterogeneous and generally characterized by cough, chest discomfort, dyspnea, low-grade fever, tiredness, weight loss, and night sweats; a completely asymptomatic course of the disease is also possible [3]. Diagnosis is based on histological evidence of non-necrotizing granulomas of the involved organs, after excluding other causes of granulomatous disorders, and in addition to typical radiological and clinical findings [4]. First-line therapy involves the use of oral corticosteroids, whereas azathioprine, methotrexate, mycophenolate mofetil, cyclosporine, cyclophosphamide, leflunomide or hydroxychloroquine can be used as second-line drugs; finally, third-line choices are represented by anti-tumor necrosis factor-a (TNF-a) antibodies, such as infliximab or adalimumab [3].

Sarcoidosis shows a bidirectional relationship with metabolic syndrome, obesity, and insulin resistance: in fact, on the one hand, sarcoidosis patients have a higher risk of developing metabolic syndrome and insulin resistance [5], while an increase in body mass index (BMI) and weight gain lead to a higher risk of developing sarcoidosis [6]. This correlation could find an explanation in the low-grade chronic inflammatory state characterizing both diseases [5], thus leading to an increase in cardiovascular (CV) risk in sarcoidosis patients [7].

In the context of dysmetabolism, triglyceride-glucose (TyG) index, which is calculated from fasting glucose and triglycerides, is interestingly emerging as a useful surrogate of insulin resistance [8–10]: it showed a higher accuracy than homeostasis model assessment of insulin resistance (HOMA-IR) in predicting metabolic syndrome [11] and a certain predictive value in terms of cardiovascular disease (CVD) [12, 13].

Moving from these premises and considering the importance of assessing metabolic risk in sarcoidosis patients, the aim of the present study was to possibly confirm the previous evidence regarding a higher prevalence of metabolic syndrome in patients with sarcoidosis, and thus to investigate the utility of TyG index in sarcoidosis clinical setting.

## Methods

### Study population

We conducted a retrospective case–control study through the enrollment of a cohort of subjects with an established diagnosis of sarcoidosis—defined as “cases” and a cohort of control subjects defined as “controls”.

Inclusion criteria for cases were the presence of a firm diagnosis of sarcoidosis according to international ATS/ERS/WASOG criteria [14] and age greater than 18 years; exclusion criteria were the presence of other causes of granulomatous disorders and age under 18 years.

For control group inclusion criteria were the absence of any pulmonary, intestinal, hepatic, rheumatological, neoplastic disease or other chronic conditions (except for metabolic comorbidities namely metabolic syndrome, dyslipidemia, arterial hypertension, and diabetes) and age greater than 18 years; exclusion criteria were the presence of any chronic condition not allowed by the inclusion criteria and age under 18 years.

Regarding “cases”, eligible subjects were consecutively selected among patients monitored at our regional Sarcoidosis Referral Centre in Bari (South Italy) from March 2020 to May 2023. In the case of controls, age- and sex-matched eligible subjects were selected among patients who were visited through our internal medicine day-service regimes from November 2022 to April 2023. Selection of cases and controls was made according to the established inclusion and exclusion criteria. For both cases, subjects with incomplete clinical, instrumental, and biochemical data were excluded. A final population of 90 consecutive cases and 90 age- and sex-matched controls was enrolled.

### Data collection

For each enrolled patient, data about age, sex, smoking habits, and comorbidities was collected; the diagnosis of metabolic syndrome was made referring to the International Diabetes Federation diagnostic criteria [15].

Waist circumference values (expressed in cm), body mass index values (expressed in Kg/m^2^), systolic, and diastolic arterial pressure values (expressed in mmHg) were also collected. The following laboratory findings were examined: fasting glucose, total cholesterol (TC), LDL-cholesterol (LDL-c), HDL-cholesterol (HDL-c), and triglycerides (TG); all these data were expressed in mg/dL.

Framingham risk score (FRS) was evaluated for CV risk assessment [16]. TyG index was also calculated using the following formula: Ln [TG (mg/ dL) × fasting glucose (mg/dL)/2] [17].

Moreover, for sarcoidosis patients’ additional information inherent to radiological classification according to Scadding criteria [18] and phenotypical stratification according to GenPhenReSa [19] were collected. Treatment regimen data, including glucocorticoid (GC) cumulative dose, when available, was obtained. Illness duration was calculated as the time elapsed between diagnosis and the enrollment data.

For both cohorts, data collection was made considering the information related to the most recent outpatient visit.

### Statistical analysis

Results were expressed as median and interquartile range (IQR). Since data did not show a normal distribution, comparisons between pairs of variables and multiple comparisons were performed using Mann–Whitney and Kruskal Wallis tests, respectively. The Spearman’s Rho test was used for correlation analysis. Contingency tables were analyzed by Chi-square test. Receiving operating characteristic (ROC) curve analysis was used to assess the predictive capacities of the TyG index. A *p*-value less than 0.05 was considered statistically significant. Statistical analysis was obtained using IBM SPSS Statistics version 26 and GraphPad Prism version 9 softwares.

## Results

### General features of the sample

90 sarcoidosis patients and 90 controls were enrolled. Body mass index (BMI) values were lacking for 37 controls. No significant differences in gender, age, BMI, and smoking status were found among the two cohort; regarding metabolic comorbidities, the prevalence of diabetes and dyslipidemia was similar among the two groups, while arterial hypertension and metabolic syndrome were more represented in the sarcoidosis group. Regarding the latter, about half of the patients took specific therapy for the disease at the time of the enrollment with a median GC cumulative dose of 5115.62 mg; interestingly, GC cumulative dose values were not significantly different depending on the presence or absence of arterial hypertension or metabolic syndrome (*p* = 0.103, and *p* = 0.417, respectively). Most of sarcoidosis patients showed pulmonary involvement with a prevalence for the second radiological stage (Table 1).

**Table 1:** General features of the sample

	Sarcoidosis	Controls	*p*-value
N° of subjects enrolled	90	90	
Male (%)	39 (43.3)	38 (42.2)	0.880_a_
*Smoking habit*			
Current (%)	5 (5.6)	13 (14.4)	0.068_a_
Never (%)	77 (85.6)	65 (72.2)	0.068_a_
Former (%)	8 (8.9)	12 (13.3)	0.068_a_
Age (median IQR)	58 (52–65)	58 (51–62)	0.209_b_
*Metabolic comorbidities*			
Diabetes (%)	23 (25.6)	18 (20)	0.374_a_
Arterial hypertensions (*%*)	46 (51.1)	32 (35.6)	0.035_a_
Metabolic syndrome (*%*)	54 (60)	31 (34.4)	< 0.01_a_
Dyslipidemia (*%*)	43 (47.8)	36 (40)	0.293_a_
Body mass index *(median IQR)*	27.29 (24.11–30)	26.5 (23–29.3)	0.184_b_
Illness duration in months (*median IQR*)	55 (24–105)		
Treated (*%*)	48 (53.3) of which		
	32 (66.6%): glucocorticoids alone		
	14 (29.1%): glucocorticoids in combination regimens		
	2 (4.1%): azathioprine		
GC cumulative dose (mg) (*median IQR*)	5115.62 (562.5–10353)		
*Arterial hypertension*			
Present	5690.62 (1500–11895)		
Absent	4853.12 (325–7845)		0.103_b_
*Metabolic syndrome*			
Present	5250 (0.00–10525)		
Absent	5076.87 (701.25–7875.00)		0.417_b_
*GenPhenReSa phenotypical stratification*			
Abdominal (%)	20 (22.2)		
Ocular-cardiac-cutaneous- central nervous system (%)	14 (15.5)		
Musculoskeletal- cutaneous (%)	1 (1.1)		
Pulmonary and intrathoracic lymph node (%)	54 (60)		
Extrapulmonary (%)	1 (1.1)		
*Scadding radiological stage*			
Stage 0 (%)	7 (7.7)		
Stage 1 (%)	15 (16.6)		
Stage 2 (%)	58 (64.4)		
Stage 3 (%)	7 (7.7)		
Stage 4 (%)	3 (3.3)		

^a^Chi square test, ^b^Mann–Whitney test

### Case–control comparison

Sarcoidosis group showed TC, LDL-c, fasting glucose, and TG values significantly higher than the control group (*p* = 0.037, *p* = 0.019, p = 0.006, and *p* = 0.001, respectively), while no significant differences were found in terms of HDL-c (*p* = 0.671). Regarding clinical data, only systolic blood pressure values were significantly higher in the sarcoidosis group than in controls (*p* = 0.035); finally, Framingham risk score showed similar values among the two groups (*p* = 0.866).

Regarding the TyG index, the analysis showed significantly higher values in the sarcoidosis group than in the control one (*p* < 0.001). Intriguingly, the data continued to be significant even if the major determinants of insulin resistance (namely metabolic syndrome and diabetes) were excluded from the analysis in both groups (*p* = 0.018) (Table 2).

**Table 2:** Case–control comparison: analysis of clinical and biochemical parameters, TyG index and Framingham risk score

	Sarcoidosis patients Median (IQR)	Controls Median (IQR)	*p*-value
*Biochemical data*			
Total cholesterol (mg/dl)	188.5 (162–223)	182 (155–205)	0.037_a_
LDL-cholesterol (mg/dl)	109 (91–138)	101 (78–124)	0.019_a_
HDL-cholesterol (mg/dl)	55 (45–68)	54 (43–68)	0.671_a_
Triglycerides (mg/dl)	111.5 (85–158)	84 (67–131)	< 0.01_a_
Fasting glucose (mg/dl)	93 (85–108)	89 (83–95)	< 0.01_a_
TyG index	8.64 (8.33–8.92)	8.22 (7.99–8.76)	< 0.001_a_
TyG index (after excluding the influence of metabolic factors)	8.33 (8.07–8.59)	8.05 (7.92–8.42)	0.018_a_
*Clinical data*			
Waist circumference (cm)	99.5 (89–110)	98 (84–104)	0.070_a_
Systolic blood pressure (mmHg)	125 (120–140)	122.5 (115–130)	0.035_a_
Diastolic blood pressure (mmHg)	80 (70–85)	80 (70–80)	0.448_a_
*Framingham risk score*	8 (4–13)	8 (4–12)	0.866_a_

^a^Mann–Whitney test

### Analysis of the TyG index in the sarcoidosis cohort

Deepening the behavior of the TyG index in the sarcoidosis cohort (Table 3), results showed a non-significant correlation with illness duration (R = 0.001, *p* = 0.989) or age (R = 0.179, *p* = 0.092). Moreover, the TyG index was independent of GenPhenReSa clinical phenotyping (*p* = 0.358), gender (*p* = 0.139) and radiological stage (*p* = 0.656). Regarding the treatment regimen, the TyG index still showed an independent trend from the need for a pharmacological treatment at the time of the enrollment (*p* = 0.093) and from the GC cumulative dose (R = 0.040, *p* = 0.682). On the other hand, the TyG index presented a significant positive correlation with waist circumference (R = 0.377, *p* < 0,001), systolic and diastolic blood pressure (R = 0.216, *p* = 0.041 and R = 0.230, *p* = 0.029, respectively); a significant positive correlation between TyG index and Framingham score was also found (R = 0.284, *p* < 0.01).

**Table 3:** Analysis of TyG index in the sarcoidosis cohort

Correlation tests (Spearman’s Rho test)	*R*	*p-value*
GC cumulative dose	0.440	0.682
Illness duration	0.001	0.989
Age	0.179	0.092
Waist circumference	0.377	< 0.001
Systolic blood pressure	0.216	0.041
Diastolic blood pressure	0.230	0.029
Framingham score	0.284	< 0.01
*Influence of phenotypical features*		
GenPhenReSa		0.358_a_
Radiological stage		0.656_a_
Treatment regimen (treated or not treated)		0.093_b_
Gender		0.139_b_

^a^Kruskal–Wallis test, ^b^Mann–Whitney test

**Fig. 1:**
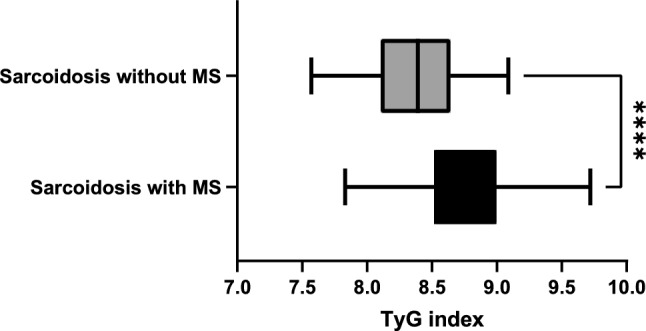
Analysis of TyG index in sarcoidosis cohort according to the presence of metabolic syndrome. *MS* metabolic syndrome; ****p value < 0.0001 with reference to Mann–Whitney test

Moreover, TyG index values were significatively higher in the subgroup of sarcoidosis with metabolic syndrome than in sarcoidosis patients without metabolic syndrome (8.77, IQR 8.52–8.98, and 8.38, IQR 8.10–8.63, respectively; *p* < 0.0001, Fig. 1). This evidence suggests that the TyG index was closely related to metabolic syndrome also in this specific cohort of patients.

### Ability of TyG index to predict metabolic syndrome and cardiovascular risk in sarcoidosis cohort

**Fig. 2:**
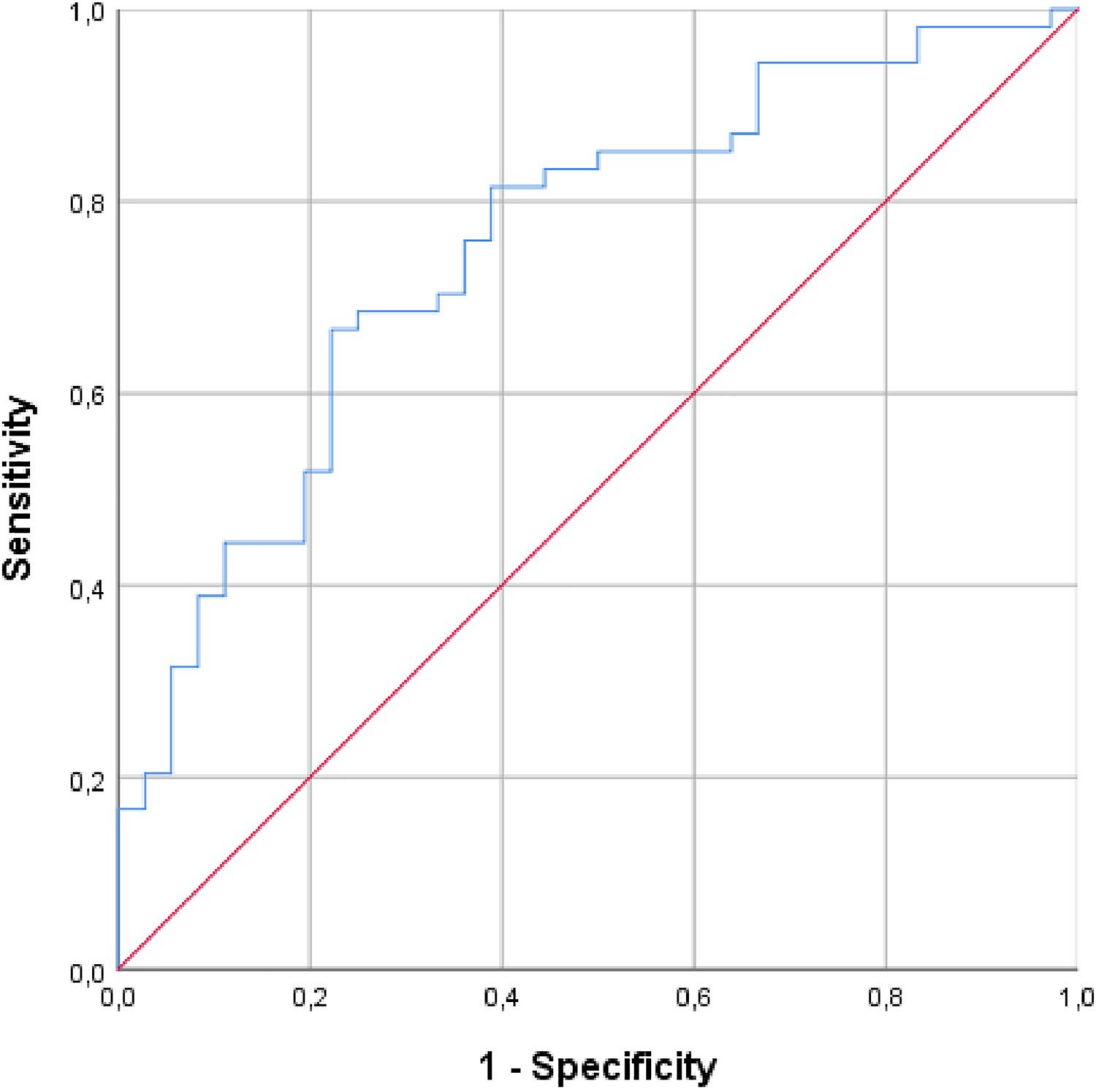
ROC curve analysis: ability of TyG index to predict metabolic syndrome in sarcoidosis cohort

ROC curve analysis was performed to test the ability of the TyG index to predict metabolic syndrome among sarcoidosis patients: according to the Youden index, a TyG index optimal cut-off value of 8.64 (66.7% sensitivity, 77.8% specificity) with an area under the curve (AUC) of 75% (95% confidence interval 65–85, *p* < 0,001) was found (Fig. 2).

**Fig. 3:**
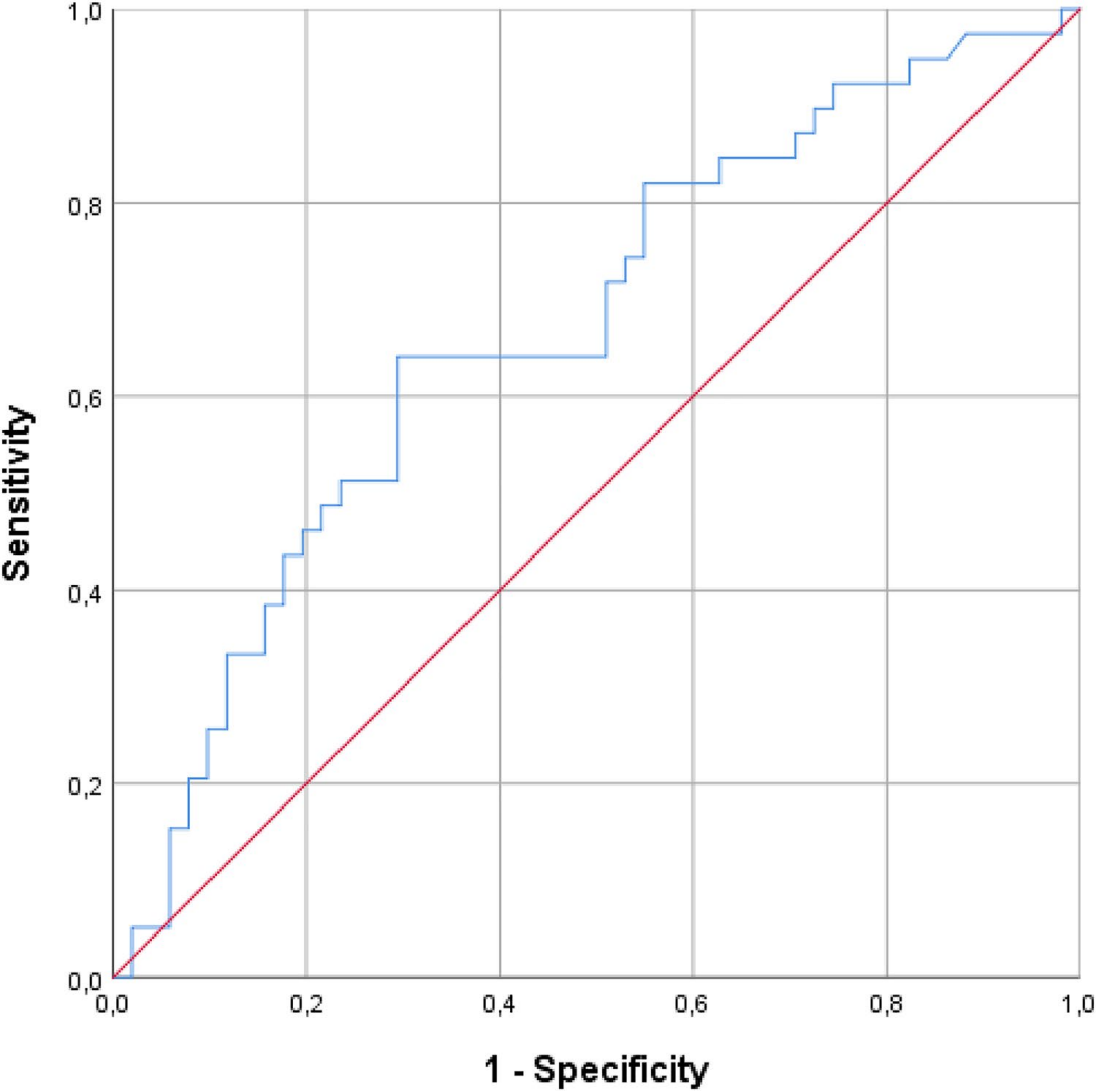
ROC curve analysis: ability of TyG index to predict a Framingham risk score ≥ 10

In the light of the positive and significant correlation between TyG index and Framingham score, ROC curve analysis (Fig. 3) was performed also to test the ability of TyG index to predict cardiovascular risk: according to Youden index, a TyG index optimal cut-off value of 8.69 (64.1% sensitivity, 70.6% specificity; AUC 67%, 95% confidence interval 55–78, *p* < 0.01) was able to identify a Framingham risk score equal or greater than 10, that is equivalent to an almost intermediate cardiovascular risk [20].

## Discussion

Sarcoidosis is a chronic inflammatory disease that can affect metabolic homeostasis, exposing patients to a higher risk of developing metabolic syndrome [5]. In the first place, both diseases share the expression of a proinflammatory cytokines pathway that includes interleukin-(IL)-6, IL-8, IL-12, and tumor necrosis factor-alpha (TNF-α): the latter is one of the most important cytokines involved in metabolic syndrome, dyslipidemia, and insulin resistance, whereas IL-6, IL-8 and IL-12 also promote metabolic syndrome [21]. In the second place, a certain susceptibility to develop dysmetabolism can be considered as an adverse effect of glucocorticoids long-term therapy [22].

For these reasons, sarcoidosis patients show a higher risk of developing atherosclerosis and CVD than the general population [7, 23]; this evidence is confirmed even when considering a subclinical level of atherosclerotic damage [24, 25].

In light of these considerations, the importance of a metabolic assessment is crucial for CVD prevention in the context of a sarcoidosis clinical setting.

The goal of our retrospective study was to possibly confirm a higher prevalence of metabolic syndrome in sarcoidosis patients and to investigate the utility and applicability of the TyG index, a surrogate of insulin resistance, to quickly identify sarcoidosis patients at high risk of metabolic syndrome.

In the first place, results showed TyG index values significantly higher in the sarcoidosis cohort than in controls, so highlighting a higher predisposition in sarcoidosis to develop metabolic syndrome. Intriguingly, the TyG index was higher in the sarcoidosis group even when subjects with diabetes and metabolic syndrome were excluded from the analysis. This evidence suggests that sarcoidosis could play an independent role in determining metabolic syndrome because of a constant, chronic low-grade inflammation. To support this hypothesis, our results showed that the TyG index was not correlated with age or illness duration, neither to phenotypical features of the disease, such as gender, different organ involvement or radiological stage. Moreover, TyG index values were not significantly different between treated and not-treated subjects and were not influenced by glucocorticoid cumulative dose: in other words, therapy seemed to be unable to shape the trend of the TyG index. This last evidence is also supported by the analysis of the trend of GC cumulative dose in two sub-cohort of sarcoidosis patients: those affected by arterial hypertension and those affected by metabolic syndrome. Our results showed that GC cumulative dose median values did not differ significantly depending on the presence or absence of these metabolic comorbidities. These results further suggest that metabolic comorbidities seemed to be influenced by the presence of the sarcoidosis itself, rather than pharmacological therapy taken by patients.

These results are coherent with a recent study that underlines how metabolic syndrome may be more frequent in sarcoidosis even before treatment is started: analyzing 133 newly diagnosed sarcoidosis patients, 133 age- and sex-matched controls, and 51 untreated rheumatoid arthritis subjects, authors found that metabolic syndrome was more common in sarcoidosis patients than controls (odds ratio 5.3, *p* < 0.001) and similar to rheumatoid arthritis group; moreover, triglycerides, glucose, waist circumference, and diastolic blood pressure values of female sarcoidosis patients were significantly higher than controls [21].

Moreover, our results showed that among sarcoidosis patients, the TyG index was significantly higher in subjects with metabolic syndrome: this finding confirms the close correlation between the TyG index and metabolic syndrome even in the specific clinical context of sarcoidosis.

Afterwards, the capacity of the TyG index to predict metabolic syndrome has been evaluated through the ROC curve analysis. With a view to improving its predictive capabilities, results suggested a potential role of the Tyg index in identifying metabolic syndrome in sarcoidosis patients: according to our findings, an optimal cut-off value of 8.64 has proved to detect metabolic syndrome similar to the previous evidence in the general population[11].

Another important consideration arises from correlation studies: indeed, the TyG index showed a statistically significant positive correlation with Framingham risk score and a potential ability to screen for cardiovascular risk: a TyG index cut-off value of 8.69 showed the capacity to detect an almost moderate cardiovascular risk in sarcoidosis group.

For all these features, the TyG index can be considered a valid tool to screen sarcoidosis patients for metabolic comorbidities, allowing physicians to detect high cardiovascular-risk patients and setting a personalized follow-up plan.

To our knowledge, our study explores for the first time the utility of the TyG index in a sarcoidosis clinical setting. Results pointed out that it can be considered a useful tool for the metabolic assessment of sarcoidosis patients because it showed a considerable capacity for screening for metabolic syndrome and cardiovascular risk. TyG index could be implemented in daily clinical practice from the perspective of a holistic evaluation of sarcoidosis patients, so considering the metabolic features of the disease that can potentially modulate its outcome. We retain that TyG index evaluation should be routinely considered for the assessment of sarcoidosis patients: in the case of high TyG index values, physicians could suspect the presence of an insulin-resistance state and consequently deepen the metabolic assessment of the patient, requiring further laboratory test such as oral glucose tolerance test. TyG index can so allow a quick achievement of crucial information to plan a careful therapeutic strategy: for example, a significant and gradual increase of TyG index in patients taking corticosteroids should alert the physician about the worsening of metabolic homeostasis, suggesting the need for introduction of steroid-sparing drugs such as methotrexate or azathioprine. From the other hand, basal high TyG index values in treatment-naïve patients could lead physicians to avoid the use of corticosteroids as the first therapeutic choice whenever possible. Finally, the TyG index could help to identify patients deserving of a screening and possible treatment of metabolic comorbidities.

The present study has some limitations. The first one is related to the retrospective design of the study which may have led to a selection bias. In detail, it could be due to the exclusion from the enrollment of subjects with incomplete collectible data and to the enrollment of the controls subjects among patients who underwent to a medical examination for a wide variety of clinical reasons; therefore, controls cohort could not be considered fully representative of the general population. For this reason, our results may have been partially affected by this methodological feature; thus, it would be desirable to confirm our findings through further perspective observational studies.

Another limitation of the study arises from the analysis of ROC curve results, which suggests the need to further deepen the TyG index behaviour in the sarcoidosis cohort, eventually improving the diagnostic accuracy of the TyG index in predicting metabolic syndrome and cardiovascular risk. In this regard, the enrollment of a greater sample size could be useful to obtain more accurate results.

With a view of an emerging need for precision medicine, clinical phenotyping could be helpful to understand prognosis and to choose the most appropriate therapeutic and follow-up planning. For example, in the context of the therapeutic field, Rana et al. found that a peripheral neutrophil-to-lymphocyte ratio could be considered a useful laboratory biomarker related to disease activity and the need for treatment [26].

However, given the high susceptibility of sarcoidosis patients to develop metabolic comorbidities, a stratification based on metabolic features should also be considered. Moreover, it can represent a challenging field of research: since sarcoidosis and metabolic syndrome partially share common pathogenetic biochemical pathways, it could be interesting to evaluate if metabolic features can somehow affect pharmacological therapeutic response. In this regard, the TyG index could be considered for further studies for the possible development of a metabolic-based phenotypical stratification model. In detail, clinicians could deepen if the metabolic homeostasis of the patient is somehow related to specific phenotypical features of the disease such as organ involvement, duration of clinical remission, response to pharmacological therapy or the impact on symptoms and quality of life. This approach eventually could help to identify a new stratification model of sarcoidosis patients capable to predict the prognostic impact and to suggest the most appropriate treatment.

In conclusion, the TyG index can be considered a promising useful tool for the metabolic assessment of sarcoidosis patients. Further studies are needed to better define how to introduce it in daily clinical practice. Moreover, the TyG index can be considered an innovative item around which new research direction can be traced, with the aim of deepening the impact of metabolic comorbidities on the prognosis of sarcoidosis patients.

## Data Availability

All data supporting the findings of this study are available from corresponding author on reasonable request.
